# Subcutaneous ketamine in the treatment of depression and suicide risk: case report.

**DOI:** 10.1192/j.eurpsy.2023.2145

**Published:** 2023-07-19

**Authors:** M. A. Cigognini, D. V. D. Meene

**Affiliations:** 1 Psychiatry, Institute and Department of Psychiatry of Clinics Hospital of the University of São Paulo Medical School, São Paulo; 2 Psychiatry, Instituto de Neurosciência & Comportamento, Blumenau, Brazil

## Abstract

**Introduction:**

Several studies have shown that ketamine, an NMDA receptor antagonist, represents a promising alternative in treating depression and suicide. The intranasal or intravenous use of ketamine, currently used, has limitations in terms of cost and complexity. The subcutaneous (SC) route may be an affordable alternative for the treatment of depression and suicidality.

**Objectives:**

To evaluate the response of SC ketamine (0,5 mg/kg) applications on depressive, anxiety, and suicide symptoms.

**Methods:**

A patient with unipolar depression and suicide attempt was submitted to 3 sessions of SC ketamine (0,5 mg/kg). The applications had 2 days of intervals. Clinical evaluations were measured by BDI, BSI, and BAI. The vital signs were monitored under 2 hours after injections and the potential side effects.

**Results:**

Changes in measurement instruments according to applications can be seen in Tab 1:

**Table:** 

	BDI	BSI	BAI
Application 1	26	14	18
Application 2	03	00	00
Application 3	02	00	00

The average measurements of vital signs during 2 hours of monitoring for each application can be seen in Tab 2:

**Table:** 

	BP	HR	RF	OX	ECG
Nine measurements (average)	123/80	78,86	17,55	99%	NP

**Conclusions:**

The use of SC ketamine showed remission in BDI, BSI and BAI, respectively demonstrated safety in use.

**Disclosure of Interest:**

None Declared

